# Insecticide-treated nets and malaria prevalence, Papua New Guinea, 2008–2014

**DOI:** 10.2471/BLT.16.189902

**Published:** 2017-09-05

**Authors:** Manuel W Hetzel, Justin Pulford, Yangta Ura, Sharon Jamea-Maiasa, Anthony Tandrapah, Nandao Tarongka, Lina Lorry, Leanne J Robinson, Ken Lilley, Leo Makita, Peter M Siba, Ivo Mueller

**Affiliations:** a Swiss Tropical and Public Health Institute, Socinstrasse 57, Basel, 4002, Switzerland.; b International Public Health, Liverpool School of Tropical Medicine, Liverpool, United Kingdom.; c Papua New Guinea Institute of Medical Research, Goroka and Madang, Papua New Guinea.; d Australian Army Malaria Institute, Enoggera, Australia.; e National Department of Health, Waigani, Papua New Guinea.; f Division of Population Health and Immunity, Walter and Eliza Hall Institute of Medical Research, Parkville, Australia.; * Deceased, formerly, Papua New Guinea Institute of Medical Research, Madang, Papua New Guinea.

## Abstract

**Objective:**

To investigate changes in malaria prevalence in Papua New Guinea after the distribution of long-lasting Insecticide-treated nets, starting in 2004, and the introduction of artemisinin-based combination therapy in 2011.

**Methods:**

Two malaria surveys were conducted in 2010–2011 and 2013–2014. They included 77 and 92 randomly selected villages, respectively. In each village, all members of 30 randomly selected households gave blood samples and were assessed for malaria infection by light microscopy. In addition, data were obtained from a malaria survey performed in 2008–2009.

**Results:**

The prevalence of malaria below 1600 m in altitude decreased from 11.1% (95% confidence interval, CI: 8.5–14.3) in 2008–2009 to 5.1% (95% CI 3.6–7.4) in 2010–2011 and 0.9% (95% CI 0.6–1.5) in 2013–2014. Prevalence decreased with altitude. *Plasmodium falciparum* was more common than *P. vivax* overall, but not everywhere, and initially the prevalence of *P. vivax* infection decreased more slowly than *P. falciparum* infection. Malaria infections were clustered in households. In contrast to findings in 2008–2009, no significant association between net use and prevalence was found in the later two surveys. The prevalence of both fever and splenomegaly also decreased but their association with malaria infection became stronger.

**Conclusion:**

Large-scale insecticide-treated net distribution was associated with an unprecedented decline in malaria prevalence throughout Papua New Guinea, including epidemic-prone highland areas. The decline was accompanied by broader health benefits, such as decreased morbidity. Better clinical management of nonmalarial fever and research into residual malaria transmission are required.

## Résumé

**Objectif:**

Étudier les changements observés à l'égard de la prévalence du paludisme en Papouasie-Nouvelle-Guinée suite à la distribution, depuis 2004, de moustiquaires imprégnées d'insecticides de longue durée et à l'introduction, depuis 2011, d'une combinaison thérapeutique à base d’artémisinine.

**Méthodes:**

Deux enquêtes portant sur le paludisme ont été menées en 2010-2011 et 2013-2014. Elles incluaient respectivement 77 et 92 villages, sélectionnés d'une manière aléatoire. Dans chacun de ces villages, tous les membres de 30 foyers sélectionnés aléatoirement ont donné des échantillons sanguins et été soumis à un test de dépistage de l'infection palustre par microscopie optique. Des données ont par ailleurs été tirées d'une enquête sur le paludisme réalisée en 2008-2009.

**Résultats:**

La prévalence du paludisme à moins de 1600 m d'altitude est passée de 11,1% (intervalle de confiance, IC, à 95%: 8,5–14,3) en 2008–2009 à 5,1% (IC à 95%: 3,6–7,4) en 2010–2011 et 0,9% (IC à 95%: 0,6–1,5) en 2013–2014. La prévalence diminuait avec l'altitude. Plasmodium falciparum n'était pas systématiquement plus courant que P. vivax, mais l'était dans l'ensemble et, dans un premier temps, la prévalence de l'infection à P. vivax a diminué plus lentement que la prévalence de l'infection à P. falciparum. Les infections palustres ont été regroupées par foyers. Contrairement à ce qu'indiquaient les résultats obtenus en 2008-2009, aucune corrélation significative entre l'utilisation d'une moustiquaire et la prévalence n'a été constatée dans le cadre des deux dernières enquêtes. Les prévalences de la fièvre et de la splénomégalie ont également diminué, mais leur corrélation avec l'infection palustre est devenue plus forte.

**Conclusion:**

La distribution à grande échelle de moustiquaires imprégnées d'insecticides a été associée à un déclin sans précédent de la prévalence du paludisme dans l'ensemble de la Papouasie-Nouvelle-Guinée, et notamment dans les régions montagneuses sujettes aux épidémies. Ce déclin s'est accompagné d'autres bienfaits sur la santé, et notamment d'une diminution de la morbidité. Il est nécessaire d'améliorer la prise en charge clinique de la fièvre non palustre ainsi que les recherches sur la transmission résiduelle du paludisme.

## Resumen

**Objetivo:**

Investigar los cambios en la prevalencia de la malaria en Papua Nueva Guinea tras la distribución de mosquiteros tratados con insecticida de larga duración, a partir de 2004, y la introducción de terapias combinadas basadas en la artemisinina en 2011.

**Métodos:**

Se llevaron a cabo dos encuestas sobre la malaria en 2010–2011 y 2013–2014. Incluían 77 y 92 aldeas seleccionadas al azar, respectivamente. En cada aldea, todos los miembros de 30 hogares seleccionados al azar dieron muestras de sangre y se evaluaron de infección de malaria con microscopio óptico. Además, se obtuvieron datos de la encuesta sobre la malaria realizada en 2008–2009.

**Resultados:**

La prevalencia de la malaria por debajo de 1600 m de altitud descendió del 11.1% (intervalo de confidencia (IC) del 95%: 8.5–14.3) en 2008–2009 al 5.1% (95% IC 3.6–7.4) en 2010–2011 y 0.9% (95% IC 0.6–1.5) en 2013–2014. La prevalencia descendía con la altitud. *Plasmodium falciparum* fue más común que *P. vivax* en general, pero no en todas partes e inicialmente la prevalencia de la infección *P. vivax* descendió más lentamente que la infección *P. falciparum*. Las infecciones de malaria se agruparon en los hogares. Al contrario que los hallazgos en 2008–2009, no se encontró ninguna asociación significativa entre el uso del mosquitero y la prevalencia en las dos últimas encuestas. La prevalencia tanto de la fiebre como de la esplenomegalia también descendió, pero su asociación con la infección de malaria se hizo más fuerte.

**Conclusión:**

La distribución de mosquiteros tratados con insecticida a gran escala se asoció con un descenso sin precedentes en la prevalencia de la malaria en toda Papua Nueva Guinea, incluidas las áreas altas con propensión a causar epidemias. El descenso estuvo acompañado de beneficios saludables más amplios, como el descenso de la morbilidad. Se requieren una mejor gestión clínica de la fiebre no relacionada con la malaria y la investigación de la transmisión residual de la malaria.

## ملخص

**الغرض:**

التحقيق في تغيرات انتشار الملاريا في بابوا غينيا الجديدة بعد توزيع الناموسيات المُعالَجة بالمبيد الحشري ذات المفعول الممتد، بدءًا من عام 2004، وطرح العلاج المجمّع المرتكز على مادة الأرتيميسينين في عام 2011.

**الطريقة:**

أُجريت دراستان استقصائيتان للملاريا في عامي 2010-2011 و2013-2014. وشملتا 77 و92 قرية تم اختيارها عشوائيًا على التوالي. في كل قرية، قدم جميع أفراد الثلاثين أسرة التي تم اختيارها عشوائيًا عينات من الدم وتم تقييمها بواسطة الفحص المجهري لمعرفة ما إذا كان هناك إصابة بالملاريا. وبالإضافة إلى ذلك، تم الحصول على البيانات من مسح الملاريا الذي تم إجراؤه في الفترة ما بين عامي 2008-2009.

**النتائج:**

انخفض معدل انتشار الملاريا في المناطق التي لا يزيد ارتفاعها عن 1600 متر من‏11.1 % (بنطاق ثقة يبلغ 95‏%: 8.5–14.3) في الفترة ما بين عاميّ 2008–2009 إلى ‏5.1‏% (بنطاق ثقة ‏95‏%: 3.6–7.4) في الفترة ما بين عاميّ 2010–2011، و0.9‏% (بنطاق ثقة 95‏%: 0.6–1.5) في الفترة ما بين عامي 2013–2014، بما يعني انخفاض انتشار المرض مع زيادة الارتفاع. كانت "المتصورة المنجلية" (الملاريا الخبيثة) أكثر شيوعًا من "المتصورة النشيطة" بشكل إجمالي ولكن ليس في كل الأماكن، كما انخفض معدل انتشار الإصابة "بالمتصورة النشيطة" بمعدل أبطأ من الإصابة "بالمتصورة المنجلية". وتم تجميع إصابات الملاريا في الأسر. وفي مقابل النتائج التي تم التوصل إليها في الفترة ما بين عامي 2008-2009، لم يتم العثور على ارتباط كبير بين استخدام الناموسية ومعدل الانتشار في الدراستين الأخيرتين. كما انخفض انتشار الحمى وتضخم الطحال ولكن أصبح ارتباطهما بعدوى الملاريا أقوى.

**الاستنتاج:**

ارتبط توزيع الناموسية المُعالَجة بالمبيد الحشري على نطاق واسع بحدوث انخفاض غير مسبوق في انتشار الملاريا في جميع أنحاء بابوا غينيا الجديدة، بما في ذلك المناطق المرتفعة المعرضة للأوبئة. ورافق هذا الانخفاض فوائد صحية أكثر، مثل انخفاض معدلات الاعتلال. ويلزم توفير إدارة سريرية أفضل للحمى غير الملارية وإجراء الأبحاث حول انتقال الملاريا المتبقية.

## 摘要

**目的:**

旨在调查自 2004 年开始分发长效驱虫蚊帐并于 2011 年开始引入青蒿素联合疗法后，新几内亚（旧称巴布亚）疟疾患病率的变化。

**方法:**

在 2010 年至 2011 年和 2013 年至 2014 期间分别进行过两次疟疾调查。这两次调查分别包括了 77 个和 92 个随机选择的村庄。 在每个村庄，30 个随机选择的家庭的所有成员均提供了血液样本，并通过光学显微镜评估疟疾感染情况。 另外，数据还来自于 2008 年至 2009 年进行的疟疾调查。

**结果:**

自 2008 年至 2009 年期间，海拔 1600 米以下的疟疾患病率从 2008 年至 2009 年的 11.1％（95％ 置信区间，CI: 8.5–14.3）降到 2010 年至 2011 年的 5.1%（95% 置信区间，CI：3.6–7.4）并在 2013 年至 2014 年降到 0.9%（95% 置信区间，CI：0.6–1.5)。患病率随着海拔高度下降而降低。 总体而言*恶性疟原虫*比*间日疟原虫*更常见,但也并非随处可见。最初，*间日疟原虫*感染的患病率比*恶性疟原虫*感染患病率降低的速度更慢。 疟疾感染聚集在家庭中。 与 2008 年至 2009 年的调查结果相反，后两次调查显示，驱虫蚊帐使用率与患病率之间没有明显的联系。 发热和脾肿大的患病率也有所下降，但其与疟疾感染的相关性日益增强。

**结论:**

大规模驱虫蚊帐的普及与新几内亚（旧称巴布亚）疟疾患病率前所未有的下降之间有相关性，包括流行病高发的高海拔地带。 这种下降伴随着范围更广的健康益处，如发病率下降。 需要对非疟疾发烧进行更有效的临床管理和研究，以降低尚有残留的疟疾传播。

## Резюме

**Цель:**

Изучить изменения в распространенности малярии в Папуа — Новой Гвинее после внедрения сеток, обработанных инсектицидами длительного действия, с 2004 года и после введения в 2011 году комбинированной терапии на основе артемизинина.

**Методы:**

В 2010–2011 гг. и 2013–2014 гг. были проведены два обследования на инфицирование малярией. Они включали соответственно 77 и 92 случайно выбранных поселка. В каждом поселке все представители 30 случайно отобранных домохозяйств сдали образцы крови, которые прошли исследование с использованием световой микроскопии на предмет заражения малярией. Кроме того, были получены результаты обследования на малярию, проведенного в 2008–2009 гг.

**Результаты:**

Распространенность малярии на высоте ниже 1600 м над уровнем моря снизилась с 11,1% (95%-й доверительный интервал, ДИ: 8,5–14,3) в 2008–2009 гг. до 5,1% (95%-й ДИ: 3,6–7,4) в 2010–2011 гг. и до 0,9% (95%-й ДИ: 0,6–1,5) в 2013–2014 гг. Распространенность инфекции снижалась по мере роста высоты над уровнем моря. Возбудитель *Plasmodium falciparum* встречался чаще, чем *P. vivax*, но не везде, и изначально распространенность инфицирования *P. vivax* снижалась медленнее, чем инфицирование *P. falciparum*. Случаи заболевания малярией концентрировались в домашних хозяйствах. В отличие от результатов, полученных в 2008–2009 гг., в двух более поздних исследованиях не было выявлено существенной взаимосвязи между использованием сеток и распространенностью инфекции. Распространенность как лихорадки, так и спленомегалии также снизилась, но их взаимосвязь с малярией стала более явной.

**Вывод:**

Широкомасштабное распространение использования сеток, обработанных инсектицидами длительного действия, было связано с беспрецедентным снижением распространенности малярии во всей Папуа — Новой Гвинее, включая районы, подверженные эпидемии. Снижение распространенности инфекции сопровождалось более широкими преимуществами для здоровья, такими как снижение заболеваемости. Требуется введение более качественных клинических протоколов ведения и лечения лихорадки, не связанной с малярией, а также проведение исследования оставшихся очагов распространения малярии.

## Introduction

Historically, malaria has been endemic throughout Papua New Guinea, except in highland areas over 1600 m, where temperatures are low and there is no stable local transmission, though imported cases and epidemics do occur.^1^^–^^3^ The causative parasites *Plasmodium falciparum*, *Plasmodium vivax, Plasmodium malariae* and *Plasmodium ovale* are transmitted by various *Anopheles* mosquito species adapted to distinct ecological niches.^4^ The epidemiology of malaria in the country and, consequently, its control are complex due to the number of parasite and mosquito species present, the variety of mosquito behaviour, the diversity of the natural environment and operational difficulties.

Since 2004, the country’s national malaria control programme has been supported by the Global Fund to Fight AIDS, Tuberculosis and Malaria. National campaigns were organized to distribute free, long-lasting insecticide-treated nets at the household level and, since late 2011, malaria rapid diagnostics tests, improved diagnostic microscopy and artemisinin-based combination therapy have increasingly been provided at public and church-run health-care facilities.^5^^,^^6^

In 2008–2009, towards the end of the first insecticide-treated net campaign, the Papua New Guinean Institute of Medical Research conducted a country-wide malaria indicator survey. It documented that 65% of households in areas covered by the campaign owned long-lasting insecticide-treated nets and that 33% of people were using them.^5^ In addition, malaria was found to be widespread, with a heterogeneous prevalence.^7^ Light microscopy diagnosis indicated that *P. falciparum* was the most common species, followed by *P. vivax*, which dominated in several locations. A few *P. malariae* infections were found but *P. ovale* was not detected in any sample. Within 1 year of the initial insecticide-treated net campaign, a significant reduction in the prevalence, incidence and transmission of malaria was documented at selected sites, even though insect vectors tended to feed outdoors.^8^ However, entomological investigations indicated that biting patterns and changes in these patterns may reduce the impact of vector control.^9^

Subsequent national malaria surveys conducted by the Papua New Guinean Institute of Medical Research in 2010–2011 and 2013–2014 to evaluate the national malaria control programme provided evidence that coverage with long-lasting insecticide-treated nets had increased.^6^^,^^10^ Here, we present data on malaria prevalence from these follow-up surveys and analyse changes relative to the baseline survey of 2008–2009.

## Methods

National malaria surveys were conducted from November 2010 to August 2011 and from November 2013 to August 2014. In both surveys, five villages were randomly selected from each of the country’s 20 provinces – organized in four regions – using a list of villages identified in the 2000 national census – the most up-to-date.^11^ Not all provinces or selected villages could be included because of problems with access and security. The pre-2012 province structure was adopted to ensure comparability over time: Hela Province was considered part of Southern Highlands Province and Jiwaka Province, part of Western Highlands Province. For each village, the survey team leader selected a random sample of 30 households using a list compiled by village leaders. All members of sampled households were eligible for inclusion. The sample size, which took into account financial and operational constraints, was adequate for detecting a 25% reduction in parasitaemia from 2008–2009 to 2010–2011 at the regional level at a 95% level of significance with 80% power. The first survey in 2008–2009, whose results are presented for comparison, included villages from only districts covered by the long-lasting insecticide-treated net campaign, but the method of selecting households and their members was identical to that in subsequent surveys.^7^

Data were collected using an adapted Malaria Indicator Survey questionnaire.^12^ Household heads provided details of each household member’s demographic characteristics and coverage by malaria interventions. A capillary blood sample was collected by finger-stick from each available, consenting household member aged over 5 months. Trained study nurses prepared one thick and one thin blood film for light microscopy. The haemoglobin concentration was measured using a portable HemoCue Hb 201^+^ photometric analyser (HemoCue AB, Ängelholm, Sweden). Symptomatic household members were offered a malaria rapid diagnostic test and treatment or referral to the nearest health-care facility, where appropriate. Axillary temperature was measured using an electronic thermometer and children aged between 2 and 9 years had their spleen palpated. Each patient’s blood sample was accompanied by information on recent travel. The locations of the survey villages were determined using a hand-held Garmin eTrex Global Positioning System device (Garmin Ltd., Olathe, United States of America).

Malaria was diagnosed by light microscopy at the Papua New Guinean Institute of Medical Research following established procedures.^7^^,^^13^ Each slide was examined independently by two microscopists, each viewing a minimum of 200 thick film fields. Slides with discordant results were examined by a third microscopist, who was certified at World Health Organization (WHO) level 1 or 2. A slide was considered positive for malaria if judged positive by at least two microscopists. For the 2010–2011 survey, additional assessments of unclear species identifications were performed at the Australian Army Malaria Institute in Australia by WHO-certified level-1 malaria microscopists. The number of parasites per 200 white blood cells was determined. The study was approved by the Papua New Guinea Medical Research Advisory Committee (MRAC no. 07.30 and no. 10.12).

### Data analysis

Measures of the prevalence of malaria infection and morbidity were age-standardized using the standard population for Asia given by the International Network for the Demographic Evaluation of Populations and Their Health (INDEPTH).^14^ Results are presented separately for villages below 1600 m in altitude and include comparisons with data from the 2008–2009 survey. For villages at 1600 m or higher, we compared data from the 2010–2011 and 2013–2014 surveys only as the 2008–2009 survey included few highland villages. To account for stratified sampling, national estimates were weighted, as described elsewhere.^5^ Splenomegaly was defined as a palpable spleen (i.e. Hackett grade 1 to 5) and anaemia was defined according to WHO recommendations, which include age-specific cut-offs and altitude corrections.^15^ Living in a high-quality house served as a proxy for having both good sanitation and a relatively high socioeconomic status, as defined elsewhere.^10^ Binary variables were compared using *χ^2^* tests and logistic regression, and non-normally distributed variables were compared using the non-parametric Mann–Whitney *U* test. Data analyses were conducted using Stata/IC v. 14.0 (StataCorp LP., College Station, USA) and the survey design was taken into account by using Statas set of commands for survey data analysis (svy).

## Results

In the 2010–2011 survey, blood samples were collected from 10 060 individuals. Of the 77 villages included, 58 (75.3%) were below 1200 m in altitude, 5 (6.5%) were between 1200 and 1599 m and 14 (18.2%), with 1539 participants, were at 1600 m or higher (Table 1). In the 2013–2014 survey, blood samples were collected from 8408 individuals. Of the 92 villages included, 66 (71.7%) were below 1200 m, 4 (4.3%) were between 1200 and 1599 m and 22 (23.9%), with 1536 participants, were at 1600 m or higher (Table 1). The small number of villages at intermediate altitudes reflects the population distribution in Papua New Guinea.^1^ The age distribution of survey participants is shown in Table 2. In the 2010–2011 survey, the participants’ median age was 19 years (interquartile range, IQR: 8–36): 14.1% (1418/10 060) were aged under 5 years and 52.8% (5290/10 028) were female. In the 2013–2014 survey, the comparable figures were 22 years (IQR: 9–38), 11.7% (985/8408) and 52.3% (4363/8348), respectively.

**Table 1:** National malaria surveys, Papua New Guinea, 2010–2014

Region and province	2010–2011 survey			2013–2014 survey	
	Villages^a^	Individuals tested		Villages^a^	Individuals tested
	No. (%)	No. (%)		No. (%)	No. (%)
**Total**	77 (100)	10 060 (100)		92 (100)	8408 (100)
**Southern Region**					
01. Western	3 (3.9)	376 (3.7)		5 (5.4)	504 (6.0)
02. Gulf	4 (5.2)	577 (5.7)		4 (4.3)	504 (6.0)
03. Central	5 (6.5)	814 (8.1)		5 (5.4)	474 (5.6)
04. National Capital District	5 (6.5)	673 (6.7)		4 (4.3)	301 (3.6)
05. Milne Bay	5 (6.5)	721 (7.2)		4 (4.3)	324 (3.9)
06. Oro	5 (6.5)	740 (7.4)		5 (5.4)	631 (7.5)
Total	27 (35.1)	3 901 (38.8)		27 (29.3)	2738 (32.6)
**Highlands Region**					
07. Southern Highlands					
Altitude ≥ 1600 m	4 (5.2)	494 (4.9)		4 (4.3)	335 (4.0)
08. Enga					
Altitude ≥ 1600 m	5 (6.5)	498 (5.0)		6 (6.5)	335 (4.0)
09. Western Highlands					
Altitude < 1600 m	3 (3.9)	295 (2.9)		2 (2.2)	164 (2.0)
Altitude ≥ 1600 m	2 (2.6)	188 (1.9)		3 (3.3)	213 (2.5)
10. Chimbu					
Altitude < 1600 m	1 (1.3)	140 (1.4)		1 (1.1)	85 (1.0)
Altitude ≥ 1600 m	3 (3.9)	359 (3.6)		4 (4.3)	253 (3.0)
11. Eastern Highlands					
Altitude < 1600 m	ND	ND		2 (2.2)	171 (2.0)
Altitude ≥ 1600 m	ND	ND		3 (3.3)	258 (3.1)
Total	18 (23.4)	1 974 (19.6)		25 (27.2)	1814 (21.6)
**Momase Region**					
12. Morobe					
Altitude < 1600 m	5 (6.5)	672 (6.7)		3 (3.3)	282 (3.4)
Altitude ≥ 1600 m	0 (0)	0 (0)		2 (2.2)	142 (1.7)
13. Madang	4 (5.2)	479 (4.8)		5 (5.4)	447 (5.3)
14. East Sepik	5 (6.5)	665 (6.6)		6 (6.5)	461 (5.5)
15. Sandaun	3 (3.9)	403 (4.0)		4 (4.3)	645 (7.7)
Total	17 (22.1)	2 219 (22.1)		20 (21.7)	1977 (23.5)
**Islands Region**					
16. Manus	5 (6.5)	629 (6.3)		5 (5.4)	547 (6.5)
17. New Ireland	5 (6.5)	708 (7.0)		5 (5.4)	494 (5.9)
18. East New Britain	5 (6.5)	629 (6.3)		5 (5.4)	409 (4.9)
19. West New Britain	ND	ND		ND	ND
20. Bougainville	ND	ND		5 (5.4)	429 (5.1)
Total	15 (19.5)	1 966 (19.5)		20 (21.7)	1879 (22.3)

ND: not determined.

^a^ For security and operational reasons, 23 villages could not be surveyed in 2010 to 2011 and 10 could not be surveyed in 2013 to 2014.

**Table 2:** Age of participants, national malaria surveys, Papua New Guinea, 2010–2014

Age, years	2010–2011 survey	2013–2014 survey
	No. (%)	No. (%)
< 1	72 (0.7)	95 (1.1)
1–4	1346 (13.4)	890 (10.6)
5–9	1621 (16.1)	1218 (14.5)
10–14	1137 (11.3)	944 (11.2)
15–19	888 (8.8)	728 (8.7)
20–39	2885 (28.7)	2510 (29.9)
≥ 40	2080 (20.7)	1967 (23.4)
Missing values	31 (0.3)	56 (0.7)
Total	10060 (100)	8408 (100)

### Malaria prevalence

Nationally, in villages below 1600 m in altitude, the age-standardized prevalence of malaria, as diagnosed by light microscopy, decreased significantly from 11.1% (95% confidence interval, CI: 8.5–14.3) in 2008–2009 to 5.1% (95% CI: 3.6–7.4) in 2010–2011 (*P* < 0.001) and to 0.9% (95% CI: 0.6–1.5) in 2013–2014 (*P* < 0.001). The prevalence of *P. falciparum* infection was higher than that of *P. vivax* infection in all surveys (Table 3). There was no evidence of *P. ovale* in any sample. For individual *Plasmodium* species, the difference in infection prevalence between subsequent surveys was significant at a *P*-value ≤ 0.001 for all comparisons except for *P. malariae* infection, for which the *P*-value for the difference between subsequent surveys was < 0.05, and for mixed *P. falciparum* and *P. vivax* infection between the 2008–2009 and 2010–2011 surveys, where the decrease was not significant.

**Table 3:** Age-standardized prevalence of *Plasmodium* infection below 1600m, by species, national malaria surveys, Papua New Guinea, 2008–2014

*Plasmodium* species	Infection prevalence % (95% CI)		
	2008–2009 survey^a^	2010–2011 survey	2013–2014 survey
	(*n* = 6424)^b^	(*n* = 8521)^b^	(*n* = 6872)^b^
All species	11.1 (8.5–14.3)	5.1 (3.6–7.4)	0.9 (0.6–1.5)
*P. falciparum*	6.6 (4.9–8.8)	3.0 (1.9–4.6)	0.8 (0.5–1.2)
*P. vivax*	3.1 (1.9–4.9)	2.0 (1.4–2.9)	0.1 (0.0–0.3)
*P. malariae*	0.3 (0.1–0.6)	0.1 (0.0–0.2)	0
*P. falciparum* and *P. vivax*	0.3 (0.1–0.5)	0.2 (0.1–0.4)	0.01 (0.0–0.08)

CI: confidence interval; *P. falciparum: Plasmodium falciparum*; *P. malariae: Plasmodium malariae*; *P. vivax: Plasmodium vivax*.

^a^ Data from the 2008–2009 survey^7^ were re-analysed by applying age-standardization.

^b^ The number of survey participants living in villages below 1600 m in altitude.

**Fig. 1:**
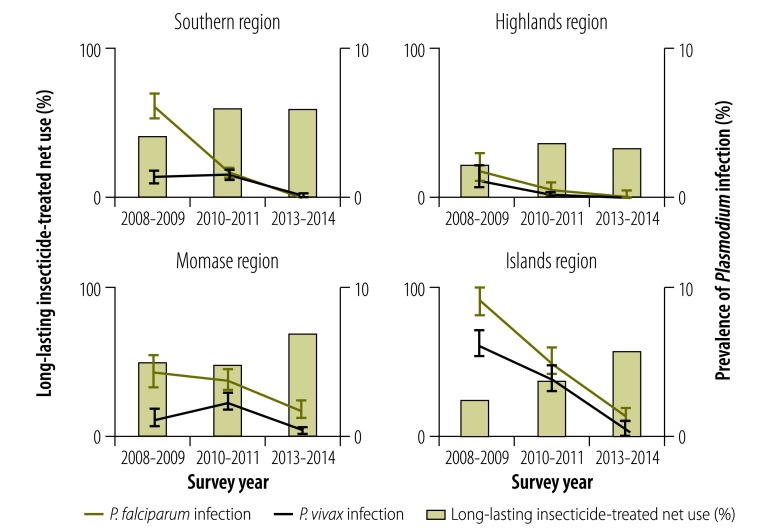
Age-standardized prevalence of *Plasmodium* infection and insecticide-treated net use, by region and survey date, national malaria surveys, Papua New Guinea, 2008–2014

**Fig 2:**
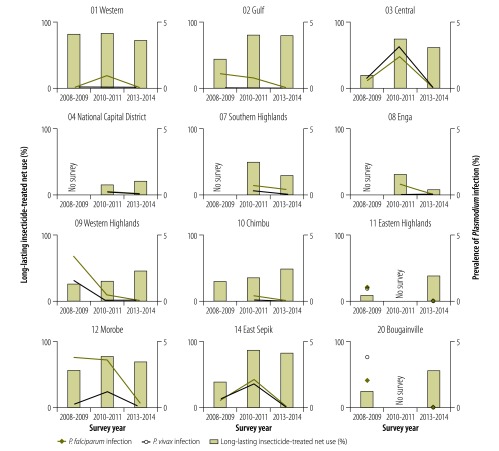
Age-standardized prevalence of *Plasmodium* infection and insecticide-treated net use in lower-prevalence areas, by province and survey date, national malaria surveys, Papua New Guinea, 2008–2014

**Fig 3:**
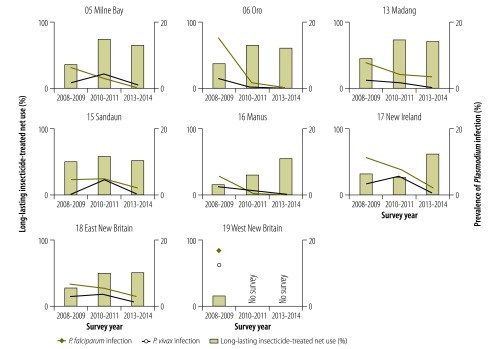
**Age-standardized prevalence of *Plasmodium* infection and insecticide-treated net use in higher-prevalence areas, by province and survey date, national malaria surveys, Papua New Guinea, 2008–2014**

Between the 2008–2009 and 2010–2011 surveys, an increase in the prevalence of *P. vivax* infection was noted in two of the country’s four regions (Fig. 1) and in several provinces (Fig. 2 and Fig. 3), which led to a decrease in the ratio of *P. falciparum* to *P. vivax* infection. However, the prevalence of infection by both species decreased in all provinces between 2010–2011 and 2013–2014. In 2013–2014, no parasites were detected in any sample from 11 of the 19 provinces surveyed (Table 4; available at: http://www.who.int/bulletin/volumes/94/10/16-189902). 

*P. falciparum: Plasmodium falciparum*; *P. vivax: Plasmodium vivax*.

Note: Error bars represent 95% confidence intervals.

*P. falciparum: Plasmodium falciparum*; *P. vivax: Plasmodium vivax*.

*P. falciparum: Plasmodium falciparum*; *P. vivax: Plasmodium vivax*.

**Table 4:** Age-standardized prevalence of *Plasmodium* infection, by province, national malaria surveys, Papua New Guinea, 2010–2014

Province and region	2010–2011 survey						2013–2014 survey				
	No. of participants	Net^a^ use (%)	Infection prevalence (%)				No. of participants	Net^a^ use (%)	Infection prevalence (%)		
			All *Plasmodium* species	*P. falciparum*	*P. vivax*				All *Plasmodium* species	*P. falciparum*	*P. vivax*
**Southern Region**											
01. Western	376	83.9	1.1	0.9	0.2		504	73.1	0.0	0.0	0.0
02. Gulf	577	80.6	0.8	0.8	0.0		504	80.0	0.0	0.0	0.0
03. Central	814	75.2	5.6	2.4	3.2		474	62.6	0.0	0.0	0.0
04. National Capital District	673	15.7	0.6	0.2	0.2		301	20.7	0.0	0.0	0.0
05. Milne Bay	721	73.5	8.3	2.9	4.2		324	63.5	0.9	0.0	0.9
06. Oro	740	65.5	2.2	1.6	0.6		631	62.5	0.0	0.0	0.0
Total	3901	60.0	3.3	1.5	1.5		2738	59.8	0.1	0.0	0.1
**Highlands Region**											
07. Southern Highlands											
Altitude ≥ 1600 m	494	50.5	1.0	0.7	0.3		335	28.9	0.4	0.4	0.0
08. Enga											
Altitude ≥ 1600 m	498	31.2	0.8	0.8	0.0		335	7.4	0.0	0.0	0.0
09. Western Highlands											
Altitude < 1600 m	295	32.3	0.5	0.5	0.0		164	47.9	0.0	0.0	0.0
Altitude ≥ 1600 m	188	29.5	0.0	0.0	0.0		213	42.2	0.0	0.0	0.0
10. Chimbu											
Altitude < 1600 m	140	41.8	0.0	0.0	0.0		85	29.9	0.0	0.0	0.0
Altitude ≥ 1600 m	359	33.5	0.5	0.5	0.0		253	53.1	0.0	0.0	0.0
11. Eastern Highlands											
Altitude < 1600 m	ND	ND	ND	ND	ND		171	38.0	0.0	0.0	0.0
Altitude ≥ 1600 m	ND	ND	ND	ND	ND		258	38.4	0.0	0.0	0.0
Total	1974	36.2	0.6	0.5	0.1		1814	32.9	0.1	0.1	0.0
**Momase Region**											
12. Morobe											
Altitude < 1600 m	672	45.7	4.8	3.6	1.2		282	77.3	0.3	0.3	0.0
Altitude ≥ 1600 m	0	NA	NA	NA	NA		142	55.0	0.0	0.0	0.0
13. Madang	479	45.7	6.3	4.5	1.8		447	70.3	3.3	3.3	0.0
14. East Sepik	665	61.9	3.9	2.1	1.7		461	83.1	0.0	0.0	0.0
15. Sandaun	403	30.1	11.0	5.0	4.9		645	52.8	3.0	2.4	0.5
Total	2219	47.0	5.9	3.6	2.1		1977	67.7	1.9	1.6	0.2
**Islands Region**											
16. Manus	629	32.0	1.8	0.7	1.3		547	56.5	0.1	0.1	0.0
17. New Ireland	708	28.4	12.9	7.9	5.8		494	62.3	2.9	2.3	0.4
18. East New Britain	629	50.8	9.2	5.4	3.6		409	51.6	4.3	3.0	1.0
19. West New Britain	ND	ND	ND	ND	ND		ND	ND	ND	ND	ND
20. Bougainville	ND	ND	ND	ND	ND		429	55.6	0.0	0.0	0.0
Total	1966	36.8	8.3	4.9	3.7		1879	56.5	1.7	1.2	0.3

NA: not applicable; ND: not determined; *P. falciparum: Plasmodium falciparum*; *P. vivax: Plasmodium vivax*.

^a^ Long-lasting insecticide-treated nets.

In highland villages at 1600 m and above, the age-standardized prevalence of malaria decreased from 0.7% (95% CI: 0.3–1.2) in 2010–2011 to 0.1% (95% CI: 0–0.7) in 2013–2014 (*P* = 0.004). In the 2010–2011 survey, the prevalence of *P. falciparum* infection was higher than that of *P. vivax* infection in highland villages: 0.6% (95% CI: 0.3–1.1) and 0.1% (95% CI: 0–0.7), respectively. In the 2013–2014 survey, the prevalence of *P. falciparum* infection was 0.1% (95% CI: 0–0.4), whereas no *P. vivax* infections were detected. Moreover, no *P. malariae*, *P. ovale* or mixed infections were found.

### Predictors of infection

**Fig. 4:**
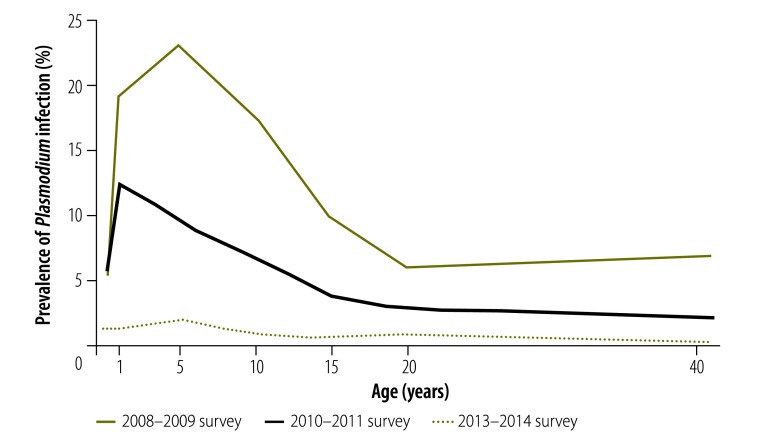
Prevalence of *Plasmodium* infection below 1600 m, by age and survey date, national malaria surveys, Papua New Guinea, 2008–2014

Note: The study age groups, as indicated in the figure, were 0–0.9, 1.0–4.9, 5.0–9.9, 10.0–14.9, 15.0–19.9, 20.0–39.9 and ≥40 years.

**Fig. 5:**
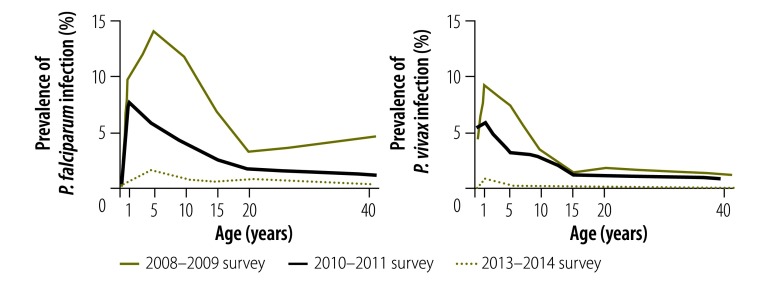
Prevalence of *P.* *falciparum* and *P. vivax* infection below 1600 m, by age and survey date, national malaria surveys, Papua New Guinea, 2008–2014

Regression analysis findings are presented in Table 5. Univariable logistic regression found that, in 2010–2011, malaria infection was significantly less likely above 1600 m (odds ratio, OR: 0.15; 95% CI: 0.06–0.38); in 2013–2014, the corresponding OR was  0.04 (95% CI: 0.00–0.33). The prevalence of infection below 1600 m was significantly lower in older individuals in all years (Fig. 4): *P* < 0.001 for the 2008–2009 and 2010–2011 surveys and *P* = 0.03 for the 2013–2014 survey. For *P. falciparum* infection, the peak prevalence shifted to a younger age between 2008–2009 and 2010–2011, but there was no corresponding change for *P. vivax* infection (Fig. 5). In 2013–2014, the difference in the prevalence of *P. falciparum* and *P. vivax* infection between age groups was not significant. However, 62.7% (95% CI: 42.2–79.4) of *P. vivax* infections and 9.1% (95% CI: 3.4–22.0) of *P. falciparum* infections occurred in children aged under 5 years.

**Table 5:** Factors associated with malaria infection, national malaria surveys, Papua New Guinea, 2010–2014

Risk factor	Risk of malaria infection		
	Univariable logistic regression analysis		Multivariable logistic regression analysis
	OR (95% CI)		aOR (95% CI)
**2010–2011 survey**			
Village at 1600 m or higher	0.15 (0.06–0.38)		0.16 (0.06–0.42)
Age < 5 years	2.98 (2.40–3.71)		2.59 (2.08–3.23)
Long-lasting insecticide-treated net use	1.38 (1.00–1.90)		1.09 (0.82–1.45)
High-quality house^a^	0.30 (0.11–0.83)		0.25 (0.08–0.79)
Percentage net use in village	1.01 (1.00–1.02)		ND
**2013–2014 survey**			
Village at 1600 m or higher	0.04 (0.00–0.33)		0.04 (0.00–0.36)
Age < 5 years	1.59 (0.74–3.44)		1.47 (0.68–3.21)
Long-lasting insecticide-treated net use	1.42 (0.83–2.45)		1.08 (0.64–1.82)
High-quality house^a^	1.00		ND
Percentage net use in village	1.01 (1.00–1.03)		ND

aOR: adjusted odds ratio; CI: confidence interval; ND: not determined; OR: odds ratio.

^a^ Living in a high-quality house served as a proxy for having both good sanitation and a relatively high socioeconomic status.

*P. falciparum: Plasmodium falciparum; P. vivax: Plasmodium vivax*.

Note: The study age groups, as indicated in the figures, were 0–0.9, 1.0–4.9, 5.0–9.9, 10.0–14.9, 15.0–19.9, 20.0–39.9 and ≥40 years.

Long-lasting insecticide-treated nets were used by 32.5% (95% CI: 27.0–38.4) of the population nationally in 2008–2009,^5^ by 48.3% (95% CI: 41.8–54.9) in 2010–2011^10^ and by 53.9% (95% CI: 49.4–58.4) in 2013–2014.^16^ In the 2008–2009 survey, a significant association was found between net use and a lower risk of malaria infection (adjusted odds ratio, aOR: 0.64; 95% CI: 0.54–0.76).^7^ However, no corresponding association was found in the 2010–2011 survey (aOR: 1.09; 95% CI: 0.82–1.45), in an analysis that adjusted for altitude, age and housing quality, or in the 2013–2014 survey (aOR: 1.08; 95% CI: 0.64–1.82), in an analysis that adjusted for altitude and age (Table 5). In 2010–2011, people living in high-quality houses were significantly less likely to be infected (aOR: 0.25; 95% CI: 0.08–0.79). Malaria cases were clustered in households. Univariable analysis found that, in 2010–2011, the odds of infection were over 26 times higher for individuals living with an infected person than for those who were not (OR: 25.65; 95% CI: 16.18–40.67); in 2013–2014, the odds were over 77 times higher (OR: 77.16; 95% CI: 41.61–143.09). In 2010–2011, 47% of malaria-infected individuals lived in a household with another infected person; in 2013–2014, the corresponding proportion was 25%.

### Morbidity

**Fig. 6:**
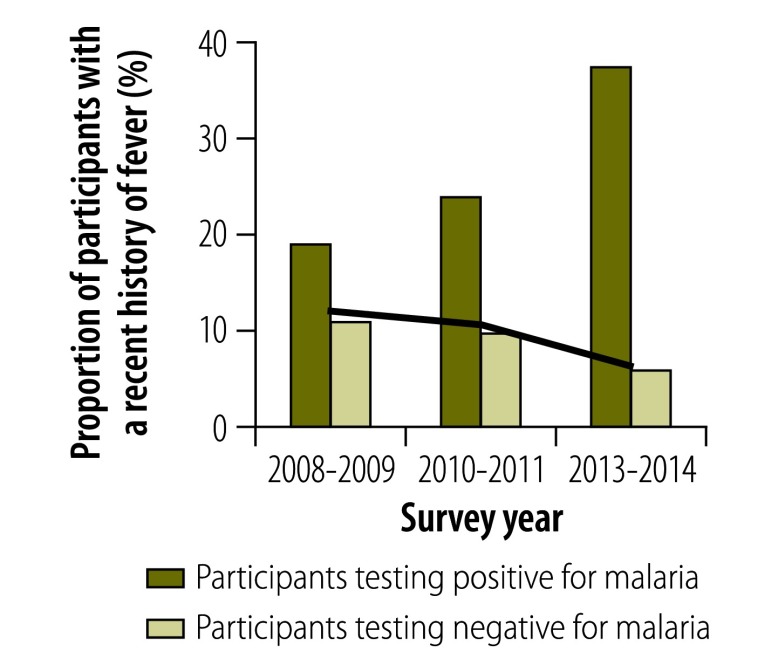
**Participants with a history of fever in villages below 1600 m, national malaria surveys, Papua New Guinea, 2008–2014**

Notes: The black line shows the proportion for each whole survey population. Differences between subsequent years in the proportion of the overall population were significant (*P* < 0.01).

**Fig. 7:**
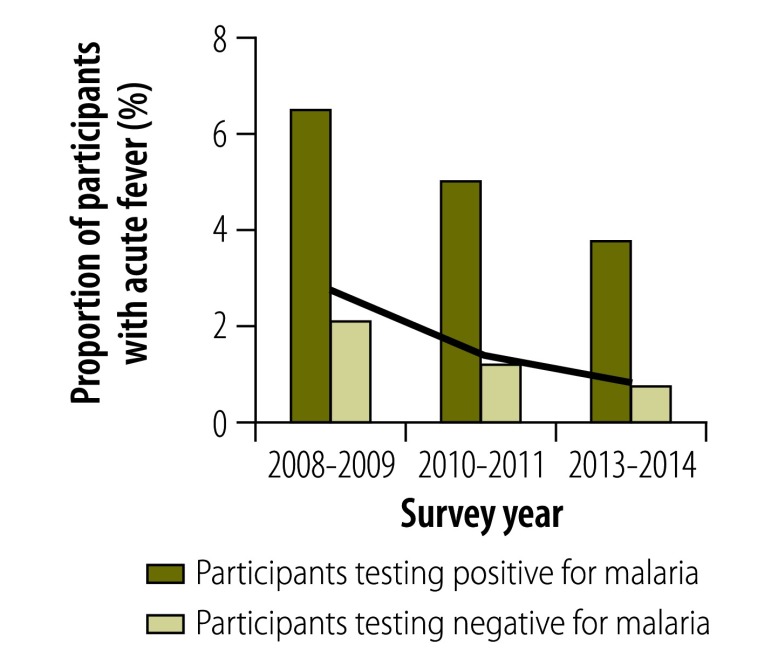
**Participants with acute fever in villages below 1600 m, national malaria surveys, Papua New Guinea, 2008–2014**

Notes: The black line shows the proportion for each whole survey population. Differences between subsequent years in the proportion of the overall population were significant (*P* < 0.01).

**Fig. 8:**
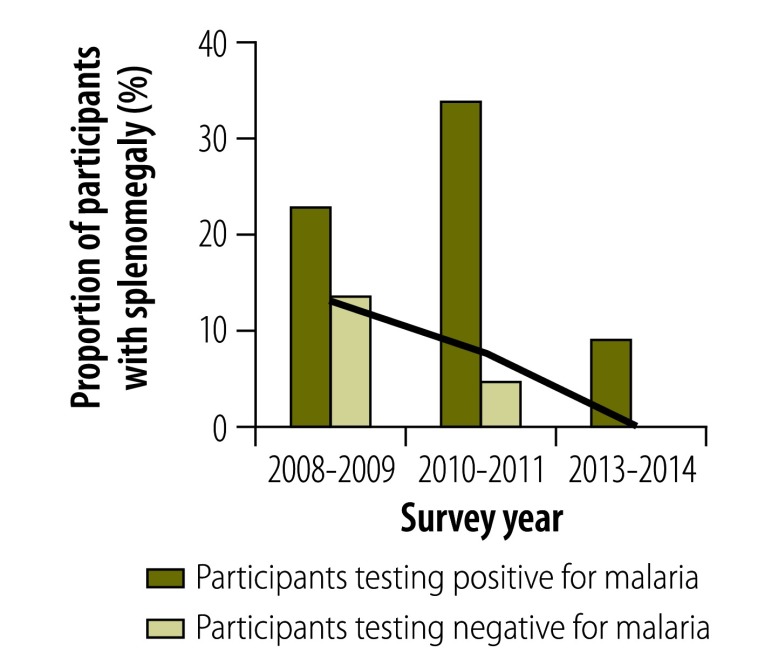
**Participants aged 2 to 9 years with splenomegaly in villages below 1600 m, national malaria surveys, Papua New Guinea, 2008–2014**

Notes: The black line shows the proportion for each whole survey population. Differences between subsequent years in the proportion of the overall population were significant (*P* < 0.01).

**Fig. 9:**
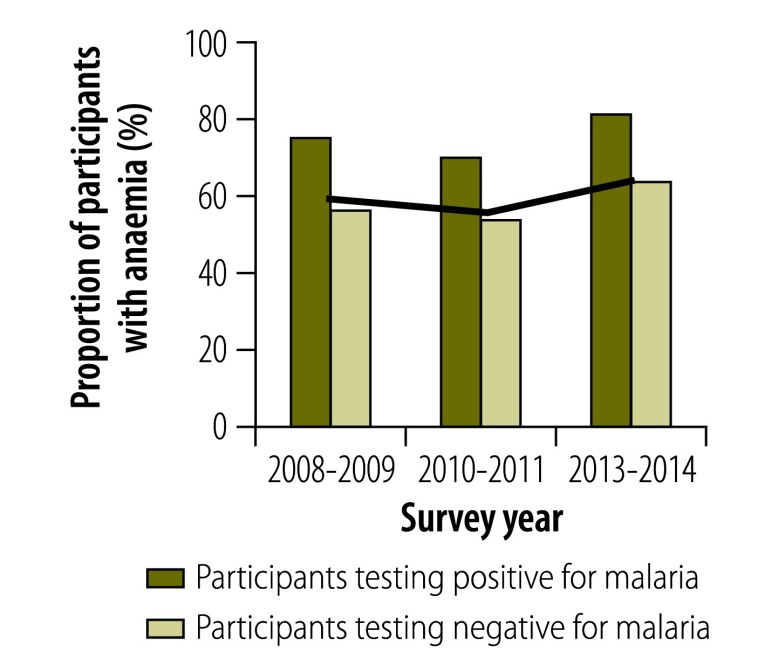
**Participants with anaemia in villages below 1600 m, national malaria surveys, Papua New Guinea, 2008–2014**

Notes: The black line shows the proportion for each whole survey population. Differences between subsequent years in the proportion of the overall population were significant (*P* < 0.01). Anaemia was defined according to WHO recommendations, which included age-specific cut-offs and altitude corrections.^15^

**Fig. 10:**
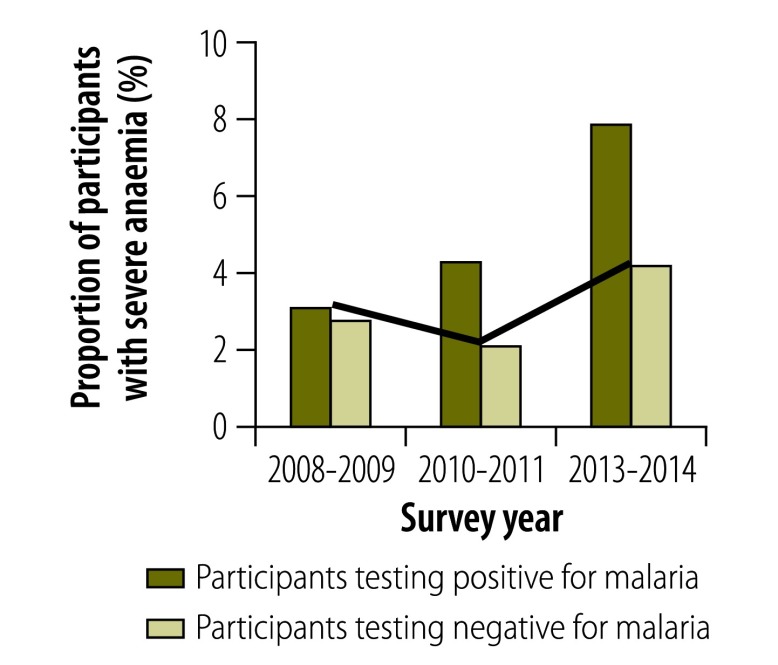
**Participants with severe anaemia in villages below 1600 m, national malaria surveys, Papua New Guinea, 2008–2014**

Notes: The black line shows the proportion for each whole survey population. Differences between subsequent years in the proportion of the overall population were significant (*P* < 0.01). Severe anaemia was defined according to WHO recommendations, which included age-specific cut-offs and altitude corrections.^15^

In all surveys, individuals infected with malaria were significantly more likely than those without to report a recent fever episode, to show symptoms of acute fever (i.e. an axillary temperature over 37.5 °C), to be anaemic or, in those aged 2 to 9 years, to have splenomegaly (*P* < 0.01 for all). Although the proportion of the population with a recent history of fever decreased over time (Fig. 6), the association between malaria infection and a recent history of fever became stronger, particularly after 2010: the OR adjusted for age was 2.05 (95% CI: 1.41–2.99) in 2008–2009, 2.57 (95% CI: 1.74–3.81) in 2010–2011 and 12.34 (95% CI: 4.56–33.33) in 2013–2014. In 2013–2014, 37.4% of all infected individuals reported a recent fever episode (Fig. 6) and 3.7% had an acute fever (Fig. 7). The prevalence of splenomegaly in participants aged 2 to 9 years also decreased over time (Fig. 8) and again the association with infection tended to become stronger: the OR adjusted for age was 4.72 (95% CI: 2.38–9.34) in 2008–2009, 10.0 (95% CI: 5.10–19.60) in 2010–2011 and 21.84 (95% CI: 5.52–88.46) in 2013–2014. The prevalence of anaemia remained high over time and increased between 2010–2011 and 2013–2014 (Fig. 9 and Fig. 10). Independent of the effect of malaria infection, in 2013–2014, anaemia was significantly associated with residing in a village below 1200 m in altitude (aOR: 8.63; 95% CI: 6.66–11.18), age under 5 years (aOR: 4.38; 95% CI: 2.96–6.46) and female sex (aOR: 1.54; 95% CI: 1.35–1.75).

## Discussion

Within 5 years, the prevalence of malaria in Papua New Guinea decreased from 11.1% to 0.9% and during 2013–2014 no parasites were detected by light microscopy in most provinces. This is a greater reduction than the 26% observed in Africa between 2000 and 2016.^17^ Moreover, the prevalence in 2014 was lower than that in other countries in the Asia–Pacific region, including the neighbouring Papua province of Indonesia.^18^^–^^21^ An initial shift towards proportionally more *P. vivax* than *P. falciparum* infections appeared to be transient and was followed by a clear reduction in both species, as observed elsewhere.^22^^,^^23^ These trends are in line with previously documented declines in malaria following the introduction of long-lasting insecticide-treated nets.^8^^,^^9^^,^^24^ Provinces in which no malaria parasites were found should not be considered malaria-free because, as parasite density decreases, an increasing proportion of infections becomes submicroscopic,^13^^,^^18^^,^^25^ particularly if transmission decreases faster than the loss of immunity. In three provinces with zero prevalence, rapid diagnostic tests found that people with fever who had not left the province had a current or recent infection. Consequently, maintaining a high level of intervention coverage is crucial for avoiding resurgence. The notion that climatic change might have increased malaria in the highlands could not be substantiated.^26^ In locations above 1600 m, malaria prevalence was lower in 2010–2011 and 2013–2014 than between 2000 and 2005.^2^ The protective effect of insecticide-treated nets in both the highlands and lowlands, from where infections are often imported,^27^ may have outweighed the impact of changing weather patterns or increased people movement. Unlike in previous years,^2^^,^^3^ *P. falciparum* was the dominant species in the highlands.

The prevalence of fever and splenomegaly declined with that of parasite infection. However, the association between infection and symptoms became stronger over time, perhaps because the proportion of microscopically detectable infections that were symptomatic increased as transmission and immunity declined. The decrease in splenomegaly was most marked, which may reflect a reduction in chronic malaria infection.^7^ On the other hand, anaemia remained common, indicating that the cause is multifactorial.^28^ Anaemia may not, therefore, be useful for monitoring rapid changes in malaria prevalence.^29^ As severe anaemia, in particular, affects children’s health and development, its causes and appropriate mitigating measures should be investigated.^28^^,^^30^

Between 2004 and 2012, the distribution of insecticide-treated nets to households was the only large-scale malaria intervention in Papua New Guinea.^10^ The baseline survey demonstrated a strong negative association between net coverage and malaria prevalence.^7^ In the absence of other factors, such as major economic developments or a prolonged drought,^9^ it is plausible that the drop in prevalence between the 2008–2009 and 2010–2011 surveys resulted from increased provision of nets and measures promoting their use. The lack of an association between net use and malaria prevalence in the last two surveys may have been due to factors such as outdoor biting, which sustained disease transmission, and the mass effect of net use on all community members.^31^ With our survey design, it was not possible to quantify the relative contributions of net use and artemisinin-based combination therapy to the reduction in prevalence. Combination therapy was introduced in November 2011 and, by late 2012, was available at approximately half of health-care facilities.^32^ Nevertheless, although the treatment’s gametocidal effect can reduce transmission from patients, its prophylactic effect is limited. Moreover, in 2014, only 45% of patients with confirmed or suspected malaria who attended health-care facilities were treated with artemisinin-based combination therapy,^33^ which corresponds to a population coverage of 19% at best. The community benefits of combination therapy can be maximized by prompt diagnosis and treatment.^34^ With decreasing malaria prevalence, clinicians across Papua New Guinea should be encouraged to administer antimalarials only to people with a positive test result, which has proven to be a safe approach,^35^ and to thoroughly investigate the causes of nonmalarial fevers. Better guidance on differential diagnosis and on fever management is warranted.^36^

We found that individuals cohabiting with another infected person were more likely to carry parasites, possibly due to similar exposure patterns. There were fewer infections in high-quality houses occupied by better-off households, possibly because of economic factors or the building’s structure or location – most high-quality houses were in urban areas. Earlier studies in Papua New Guinea found conflicting evidence of the impact of housing, namely raised structures, on mosquito exposure.^37^^,^^38^ As indoor exposure to malaria vectors has been reduced by nets, people’s outdoor behaviour may be an increasingly important determinant of exposure as many vectors tend to bite outdoors.^9^^,^^39^ The investigation of residual malaria transmission is crucial for eliminating the disease and should take into account human and mosquito behaviour patterns, including the distribution of different *Anopheles* populations, their biting preferences and their susceptibility to interventions.^8^^,^^9^^,^^37^

In our study, we used age-standardization to account for differences in the age-composition of participants between surveys and between participants who gave blood samples and the general population. However, as the 2008–2009 survey included only districts where nets were distributed, the national prevalence of parasite infection may have been underestimated. Data from sentinel sites showed that the prevalence after net distribution was 4.8% compared with 15.7% before.^8^ In addition, the estimated prevalence in the 2010–2011 and 2013–2014 surveys may have been too low because, due to security concerns, they excluded West New Britain Province, where the prevalence is traditionally high.^40^

In conclusion, increased use of long-lasting insecticide-treated nets in Papua New Guinea was associated with a rapid and significant decline in malaria prevalence – the lowest prevalence ever recorded was in 2013–2014. The decline also occurred in the epidemic-prone highlands. Light microscopy showed that *P. falciparum* remained more common than *P. vivax*. Declining prevalence was accompanied by broader health benefits, such as decreased morbidity. However, nonmalarial fever now requires better clinical management. Research into the drivers of residual malaria transmission and the burden and role of submicroscopic parasite infection are crucial for better targeting of interventions and for eliminating the disease.
